# Time delays in diagnosis of pulmonary tuberculosis: a systematic review of literature

**DOI:** 10.1186/1471-2334-9-91

**Published:** 2009-06-11

**Authors:** Chandrashekhar T Sreeramareddy, Kishore V Panduru, Joris Menten, J Van den Ende

**Affiliations:** 1 Department of Community Medicine, Manipal Teaching Hospital, Manipal College of Medical Sciences, Pokhara, Nepal; 2 Department of Internal Medicine, Manipal Teaching Hospital, Manipal College of Medical Sciences, Pokhara, Nepal; 3 Department of Public Health, Institute of Tropical Medicine, Antwerp, Belgium; 4 Department of Clinical Sciences, Institute of Tropical Medicine, Antwerp, Belgium; 5 Current address: Department of Community Medicine, Melaka-Manipal Medical College, Jalan Batu Hampar, Bukit Baru, Melaka, Malaysia; 6 Current address: Department of Respiratory Medicine, Ministry of Health, Brunei Darussalam

## Abstract

**Background:**

Delay in diagnosis of pulmonary tuberculosis results in increasing severity, mortality and transmission. Various investigators have reported about delays in diagnosis of tuberculosis. We aimed at summarizing the data on these delays in diagnosis of tuberculosis.

**Methods:**

A systematic review of literature was carried out. Literature search was done in Medline and EMBASE from 1990 to 2008. We used the following search terms: delay, tuberculosis, diagnosis, and help-seeking/health-seeking behavior without language restrictions. In addition, indices of four major tuberculosis journals were hand-searched. Subject experts in tuberculosis and authors of primary studies were contacted. Reference lists, review articles and text book chapters were also searched. All the studies were assessed for methodological quality. Only studies carried out on smear/culture-positive tuberculosis patients and reporting about total, patient and health-care system delays were included.

**Results:**

A total of 419 potential studies were identified by the search. Fifty two studies qualified for the review. The reported ranges of average (median or mean) total delay, patient delay, health system delay were 25–185 days, 4.9–162 days and 2–87 days respectively for both low and high income countries. Average patient delay was similar to health system delay (28.7 versus 25 days). Both patient delay and health system delay in low income countries (31.7 days and 28.5 days) were similar to those reported in high income countries (25.8 days and 21.5 days).

**Conclusion:**

The results of this review suggest that there is a need for revising case-finding strategies. The reported high treatment success rate of directly observed treatment may be supplemented by measures to shorten the delay in diagnosis. This may result in reduction of infectious cases and better tuberculosis control.

## Background

Tuberculosis (TB) remains a major global public health problem [1]. It is estimated that about one-third of the world's population is infected with Mycobacterium tuberculosis [2]. Among the communicable diseases, TB is the second leading cause of death worldwide, killing nearly two million people each year [3]. Early diagnosis of the disease and prompt initiation of treatment are essential for an effective tuberculosis control program. Delay in the diagnosis may worsen the disease, increase the risk of death and enhance tuberculosis transmission in the community [4].

In developing countries, emphasis is laid on passive case finding which is based on diagnosing infectious cases of tuberculosis mainly through direct microscopy of sputum specimens obtained from persons who present themselves to the health facilities [5]. But passive case finding is known to be influenced by factors such as patient motivation, the degree of diagnostic suspicion by health workers and the quality of laboratory facilities [6]. The directly observed treatment with short course (DOTS) strategy of the national TB control programs emphasizes passive case finding which could result in a delayed diagnosis [7]. These delays may be attributed to both patients as well as the health care system. The patients may delay in seeking help or the health care system may delay in suspecting and investigating for TB. It is important to identify such delays in order to undertake measures to make the TB control programs more effective.

Recent studies have shown that health-care seeking behavior of chest symptomatics is inadequate [8] and health services-related factors have led to sub-optimal utilization of diagnostic processes [9]. TB control programs of the high TB burden countries need universal coverage of DOTS strategy, political commitment and increased case detection rate [10]. Various studies have reported about the delays in diagnosis of tuberculosis in different groups of populations. It is not clear if active case finding would improve the efficacy of the current global DOTS strategy [11].

We carried out a systematic review to summarize the evidence from studies reporting time delays in diagnosis of pulmonary TB among chest symptomatics. The aims of this review were: 1) to summarize the patient, health-care system and total delays to diagnosis of pulmonary tuberculosis; 2) to compare the delay in diagnosis between low and middle income countries [LMICs] and high income countries [HICs].

## Methods

### Search criteria

We searched the following databases for primary studies: PubMed (http://www.ncbi.nlm.nih.gov/sites/entrez?db=pubmed, from January 1990 to December 2008), EMBASE (http://www.embase.com/search, from January 1990 to December 2008), HINARI (http://www.who.int/hinari/en/, from January 1990 to December 2008) and Cochrane infectious disease group trials register http://www.update-software.com/clibng/cliblogon.htm. We used the search terms "delay", "diagnosis", "pulmonary tuberculosis", and "health care seeking behavior". The search was done without language restrictions, except for the condition of an abstract being in English. We also hand searched the indices of the following journals: International Journal of Tuberculosis and Lung Disease; Tuberculosis; Tubercle and Lung Disease and BMC Infectious Diseases. Additional studies were identified by contacting the authors of the primary studies and experts in tuberculosis, by referring to the reports of World Health Organization (WHO), and by searching the reference lists of primary studies, review articles, editorials and text book chapters.

### Assessment of the studies

The list of titles and abstracts we identified were screened independently by two of the authors/reviewers (CTS and PVK). We included those studies which had reported the patient, health system and total delay in the diagnosis of pulmonary tuberculosis made by either sputum/culture positivity. The selected studies were assessed for methodological quality by two of the authors (CTS and PVK). The reviewers were not blinded to the names of authors of selected studies.

### Exclusion criteria

We excluded studies which described extrapulmonary tuberculosis only, studies which did not report the delays in diagnosis separately for pulmonary and extrapulmonary TB, studies assessing health care seeking behavior only and pure qualitative studies. Although data on patient, health system and total delays were required, a few studies which lacked total delay were not excluded: we summed up the patient and health system delay to obtain total delay.

### Data extraction and analysis

Two reviewers (CTS and PVK) extracted the data from included studies: duration of patient, health system and total delay. The duration of delay reported in weeks or months was transformed in number of days wherever necessary. Additional data consisted of country, survey year, and type of first contact, type/s of health-care facilities where the study was carried out (study setting), study design, and sample size. As time duration does not generally follow normal distribution we favoured median duration, but if median lacked, mean was considered. The definitions for the delays were not uniform in the included studies. Most studies used for total delay the time until the diagnosis, some until treatment. In such a case we considered the delay until diagnosis as it was reported by the authors or we calculated delays until diagnosis. We categorized the selected studies as those carried out in LMIC and HIC according to the definition given by the World Bank. The average of the three types of delays was calculated from the extracted data according to the two groups of countries.

### Operational definitions

World Bank definitions for LMICs and HICs: World Bank has classified economies based on gross national income (GNI) per capita during the year 2007. According to this the countries are grouped as follows: low income: $935 or less, low middle income $936 to $ 3705, upper middle income: $3707 to $ 11,455 and high income: $ 11, 456 or more. Patient delay was defined as the period from onset of the first symptom(s) possibly related to pulmonary TB to the date when the patient first contacted any type health care service (formal or informal).

**Figure 1:**
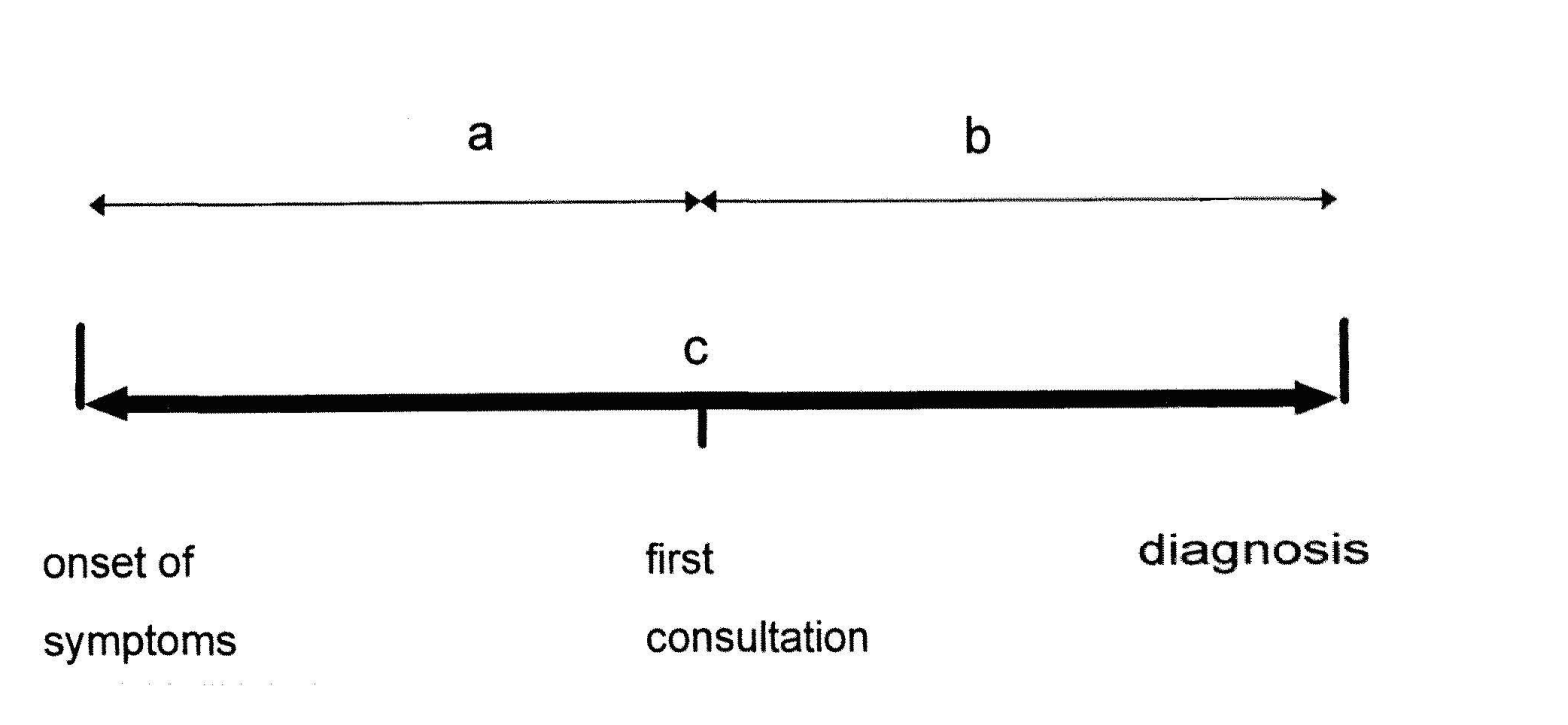
**Conceptual framework of the study and definitions of delays (Modified and adapted from Yimer et al) **[12]. a = patient delay – the time from onset of symptoms to until the patients see the first health care practices. b = health care system delay – the time from first health care seeking for diagnosis until the diagnosis made. c = total delay (a + b).

Health system delay was defined from the date of the patient's first contact with the health care service to the date of diagnosis. Total delay was defined as the sum of patient delay and health system delay [12]. (Figure 1)

We summarized the average patient, system and total delays using box-plots, showing medians, interquartile ranges (25th and 75th percentile), and ranges (minimum and maximum). We estimated median differences between low and high income countries in delays, together with 95% confidence intervals (using the bootstrap method). Although aim of our study was primarily descriptive, we tested for differences between low and high income countries using Wilcoxon's rank-sum tests. As the outcome of interest was to estimate delay in diagnosis, rather than significant effects, publication bias due to non-significant studies not being published was unlikely to occur.

## Results

### Search results

**Figure 2:**
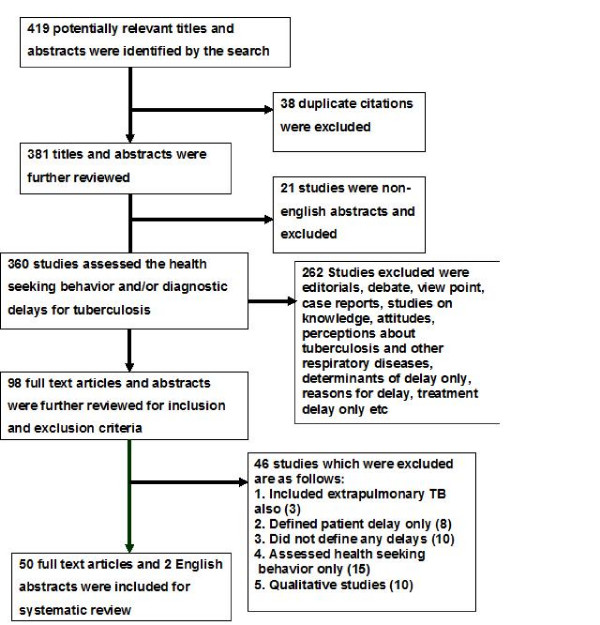
**Search results and selection of studies for systematic review**.

The flow diagram about the results of search and their inclusion for the review are shown in figure 2. Our search strategy identified a total of 419 potential studies. Three hundred and sixty studies reported about health seeking behavior and/or delays and had an English abstract. Two hundred and sixty two studies did not provide adequate information about delays (editorials, comments or case reports). Of the remaining 98 studies, 50 full-text articles and two English abstracts corresponded to the inclusion/exclusion criteria. Reasons for exclusion were qualitative methodology, inappropriate definition of delays, study of health care seeking behavior only and extrapulmonary TB. (Figure 2)

**Figure 3:**
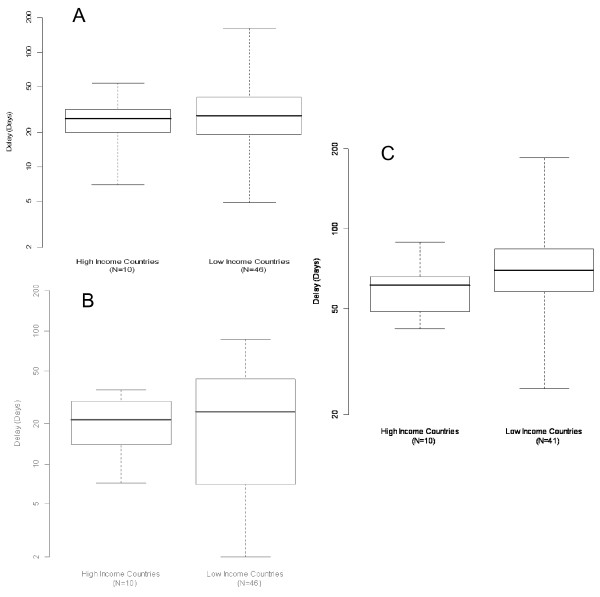
**Summary^† ^of patient, health care system and total delays in LMIC and HIC**. **3. a. Patient Delay**. Note: Difference in medians (95% confidence interval): 1.5 (-7.5, 12). P-value for Wilcoxon Rank-Sum-Test = 0.637. ^† ^Boxplots showing median (central line), interquartile range (box) and range (whiskers). **3. b. Health system delay**. Note: Difference in medians (95% confidence interval): 3.2 (-8.5, 15.3). P-value for Wilcoxon Rank-Sum-Test = 0.684. **3. c. Total delay**. Note: Difference in medians (95% confidence interval): 8.5 (-2, 25). P-value for Wilcoxon Rank-Sum-Test = 0.204.

Out of the 52 retained studies six were retrospective analyses, 12 were longitudinal patient recruitment studies with structured interview of the patients and 26 studies were designed as cross-sectional surveys with patient interviews. For the remaining eight studies, design was either not clearly stated or mixed methods was used. A WHO multi-centre study carried out in seven countries of the eastern Mediterranean region used a cross-sectional survey design and was considered as one study. Forty six studies were carried out in LMICs and 10 in HICs. Details of the studies from LMICs and HICs and their main results are described in table 1[13-51] and table 2[52-61]. The summary of delays for diagnosis of pulmonary tuberculosis in LMICs and HICs are shown in figures 3a, b and 3c.

**Table 1:** Time delays in diagnosis among pulmonary tuberculosis patients in Low and middle income countries

Year of Study	Country	Study Design	Study setting	Patient delay	Health system delay	Total delay	Reference
1997	Vietnam	Cross-sectional, Interview	District TB Units	3 w	7 w	9.9 w	[13]
1999	South Africa	Cross-sectional, interview	Hospitalized patients	4 w	4 w	10 w	[14]
1998	Tanzania	Cross-sectional, interview	Government health facilities	162 d	21 d	185 d	[15]
2003	Ethiopia	Cross-sectional, interview	TB management Units	15 d	61 d	80 d	[16]
2002	China	Longitudinal recruitment, interview	County TB Dispensaries	12.5 d	16 d	25 d	[17]
2001	China	Cross-sectional, patient records & interview	All types of health facilities	12.5 d	2 d	57 d	[18]
1997–1998	India	Cross-sectional	All types of health facilities	20 d	23 d	60 d	[19]
1998–2000	Thailand	Longitudinal recruitment	Tertiary hospital	13.3 d	8.2 d	-	[20]
1996	Vietnam	Cross-sectional, interview	Government health facilities	5.8 w	6.1 w	11.9 w	[21]
2002	Uganda	Cross-sectional, interview	TB treatment centres & hospitals	1 w	9 w	12 w	[22]
2000–2001	Nigeria	Longitudinal recruitment, interview	Tertiary hospital	8 w	1 w	10 w	[23]
2002	Vietnam	Cross-sectional, interview	District TB units	3 w	1 w	4 w	[24]
2004	China	Cross-sectional, interview	TB dispensaries	30 d	24 d	65 d	[25]
2003	India	Cross-sectional, TB records and interview	DOTS clinics	21 d	7 d	32 d	[26]
2003	India	Cross-sectional, interview	Government health facilities	28 d	28 d	62 d	[27]
2003–2004	Thailand	Longitudinal recruitment, interview	Community and tertiary hospital	4.4 w	2.8 w	9.4 w	[28]
2001	Ethiopia	Cross-sectional, interview	Public health centres	60 d	6 d	64 d	[29]
1993–1994	Botswana	Cross-sectional, TB records & interview	TB clinics	3 w	8 w	12 w	[30]
1997–1999	Nepal	Longitudinal recruitment & interview	Government health facilities	0.7 m	1.5 m	2.8 m	[31]
1995	Ghana	Cross-sectional, interview	Chest clinic of a teaching hospital	4 w	8 w	12 w	[32]
1997	Malaysia	Cross-sectional, Interview	University Hospital	2 w	7 w	12.5 w	[33]
2003–2004	Malaysia	Cross-sectional, Interview	TB clinics	30 d	22 d	-	[34]
1999	Turkey	Cross-sectional, Interview	Hospitalized patients	26.9	13.1 d		[35]
2003	Bolivia	Cross-sectional, interview	TB clinics	3.6 w	6.2 w	12.9 w	[36]
1997	Gambia	Cross-sectional, interview	All types of health centres	0.7 w	10.8 w	11.5 w	[37]
2004	Turkey	Cross-sectional, interview	Tertiary care hospital	46.4 d	30.3 d	-	[38]
2001	Turkey	Cross-sectional, interview	Tertiary care hospital	31.4 d	25.4 d	-	[39]
2006	China	Historical cohort, interview	-	19 d	5 d	31 d	[40]
1994	Malaysia	Longitudinal recruitment, interview	Tertiary care hospital	15 d	35 d	90 d	[41]
1996	Mongolia	Cross-sectional, Interview	Specialist TB centres	29 d	35 d	78 d	[42]
2008	China	Retrospective analysis of computerized patient records	County TB centres	60 d	4 d	71 d	[43]
2008	Argentina	Cross-sectional survey, Interview	Public health facilities	59 d	33 d	92 d	[44]
2007	Bangladesh	Cross-sectional survey	Public health facilities	50 d	11 d	60 d	[45]
2003/4	Pakistan	Cross-sectional survey, interview	Chest clinics	9 d	87 d	91 d	[46]
2003/4	Iran	Cross-sectional survey, interview	Public health facilities	24 d	42 d	88 d	[46]
2003/4	Somalia	Cross-sectional survey, interview	DOTS centres	53 d	7 d	58 d	[46]
2003/4	Egypt	Cross-sectional survey, interview	Public health facilities	12 d	18 d	42 d	[46]
2003/4	Iraq	Cross-sectional survey, interview	Tertiary care hospital	31 d	2 d	36 d	[46]
2003/4	Yemen	Cross-sectional survey, interview	Public health facilities	28 d	4 d	35 d	[46]
2002–2004	Syria	Cross-sectional interview	DOTS centres	52.7 d	27.6 d	77.6 d	[46]
2008	South Africa	Cross-sectional, interview	Hospital-based	14	30	60	[47]
2003	Syria	Prospective recruitment	DOTS Clinic	31	12	55	[48]
2005	Kenya	Cross-sectional interview	TB/Chest clinic	42	2	44	[49]
2003	Columbia	Cross-sectional interview	Chest/TB Clinics	30	60	120	[50]
2006	Pakistan	Cross-sectional	Chest clinic at Teaching hospital	33	60	90	[51]

d-days, w-weeks, m-months

**Table 2:** Time delays (in days) in diagnosis among pulmonary tuberculosis patients in High income countries

Year of Study	Country	Study Design	Study setting	Patient delay	Health system delay	Total delay	Reference
1992	Korea	Longitudinal Recruitment	Health centres	54 d	14 d	60 d	[52]
1997	Japan	Longitudinal recruitment, interview	Tertiary hospital	21 d	7.2 d	42 d	[53]
2000–2001	USA	Longitudinal recruitment, interview	Tertiary care hospital	32 d	26 d	89 d	[54]
1994	USA	Longitudinal recruitment, interview	All types of health facilities	25 d	15 d	57 d	[55]
2003–2004	Norway	Retrospective analysis	National TB registry	28 d	33 d	63 d	[56]
2001–2002	UK	Retrospective analysis	TB register	34.5 d	29.5 d	78 d	[57]
2003	Taiwan	Cross-sectional, interview	National TB register	7 d	23 d	44 d	[58]
2004	Hong Kong	Longitudinal recruitment, Interview	TB units	20 d	20 d	49 d	[59]
1985–1998	Australia	Retrospective analysis	TB registry	30 d	11 d	66 d	[60]
2003	Italy	Retrospective analysis	Disease surveillance data	7 d	36 d	65 d	[61]

### Time delays to diagnosis

The reported average (median or mean) total delay, patient delay, health system delay for diagnosis of tuberculosis were ranging from 25 to185 days, 4.9 to 162 days and 2 to 87 days respectively in both LMICs and HICs together. The overall average patient delay was similar to health system delay (31.03 versus 27.2 days). This was not different when analysed separately for LMICs and HICs (p-value 0.506). In HICs the average health system delay was more than three weeks.

### Patient delay

Among the LMICs patient delay varied from 4.9 days in Gambia [37] to 162 days in Tanzania [15]. The average patient delay in LMICs was 31.7 days. Among the HICs patient delay ranged from 7 days in Italy [56] and Taiwan [53] to 34.5 days in UK [52]. The average patient delay in HIC was 25.8 days, which is similar to that of LMICs (32.2 days, p-value 0.637). (Figure 3a).

### Health care system delay

Among the LMICs health care system delay ranged from the shortest of 2 days in China [18] to a longest of 87 days in Pakistan [46]. The average delay was 28.4 days. Among the HICs health care system delay ranged from a shortest of 7.2 days in Japan [48] to a longest of 36 days in Italy [56] with an average delay of 21.5 days. The difference in average health care system delay between LMICs and HICs was not statistically different (p-value 0.684). (Figure 3b)

### Total delay

Among LMICs the total delay ranged from a shortest of 25 days in China [17] to a longest of 185 days in Tanzania [15], with an average of 67.8 days. Among the HICs total delay ranged from a shortest of 42 days in Japan [48] to 89 days in USA [49], with an average of 61.3 days. The difference in average total delay between LMICs and HICs was not statistically different (p-value 0.204). (Figure 3c)

## Discussion

### Main results and comparisons with existing literature

Our systematic review of literature of 52 studies on delays in diagnosis of tuberculosis showed that there is a considerable time delay between the onset of symptoms of pulmonary tuberculosis and diagnosis. However comparison of these (patient, health system and total) delays to diagnosis between LMICs and HICs were not significantly different. Another systematic review of the same topic reported about various patient factors related to delayed diagnosis of tuberculosis. However this study did not discuss about the implications of time delays in diagnosis of TB on its control [62].

Nothing is known about an acceptable time delay from the onset of symptoms until the diagnosis is made. It is generally considered that for an effective TB control, the target for patient delay should not be more than 2 or 3 weeks [63]. Our analysis found that in both LMICs as well as HICs patient delay far exceeded this limit by about 10–15 days. As far as health system delay is considered it should be only a few days. If tuberculosis is suspected sputum test should be requested and processed immediately. Our analysis found that health system delay was about four weeks in LMICs and three weeks in HICs. Therefore, in an ideal world, the total delay to diagnosis should not be more than 3–4 weeks for most of the smear-positive TB patients. From our analysis it was observed that this limit was exceeded by about four weeks in HICs and five weeks in LMICs.

### Weaknesses of our methodology

The studies reported varying time delays. One of the reasons for such variations was the different definitions used for the first contact with the health system (any type formal or informal). For our analysis we used contact with any type of health system. This resulted in wide disparities in the patient delay and health system delays from studies carried out in different countries. For example in Gambia there was yawning gap between the patient delay (5 days) and health system delay (76 days) as this study considered contact with traditional healer for the estimation of health system delay [37].

Despite carrying out a comprehensive search some studies may have been missed to be included for this systematic review. We did not carry out a sub group analysis to decrease the heterogeneity between various studies. Since we excluded non-English abstracts we may have missed some non-English full-text articles. If studies which did not find a longer time delays in diagnosis may not have been published which would have resulted in publication bias. Some studies reported mean delay instead of median delay, which may have resulted in difficulties in estimating the average time delay to diagnosis in our analysis.

### Impact of our results

The delays have importance in transmission dynamics of tuberculosis and TB prevention and control strategies, hence also on the current global strategy for tuberculosis control. It has been reported that the global DOTS strategy for TB has improved the overall treatment success rate [7]. However, rapid geographic coverage by DOTS has not resulted in improved case finding [64]. There are further reports that current case detection rates are low and there is a need for developing new case finding strategies to reach the targets under global DOTS strategy [65]. A study from India has reported that improving the case finding methods may save 10 times more patients than by the DOTS [66]. The time delays to diagnosis identified by this review confirm these suggestions. A recent analysis of TB transmission dynamics and delay has stressed that time delays to diagnosis are the most important obstacles to the control of the TB epidemic [67]. It is important to investigate the causes of such delays to diagnosis. Most studies have reported about the patient factors responsible for the delay and have suggested improvement of patient knowledge/awareness [63]. Another review has concluded that the crux of the problem is the repeated visits to a healthcare provider for diagnosis at the same level of the healthcare system [62]. The health system may be failing to suspect and diagnose TB early enough to reduce the delay in diagnosis. From our analysis it is clear that both patient and the health system are to blame as both patient and health care system delays were longer than acceptable limits.

### Future research

Our understanding of the health system factors responsible for the time delay in diagnosis is limited. Further research is needed to analyze the health system factors responsible for delay in diagnosis of TB. It has been suggested that active case finding strategies (in target groups, symptom screening, house-to-house case search, and contact investigation) rather than just passive case finding may be helpful to reduce the delays in diagnosis [68]. It may also be important to choose appropriate case finding strategies based on the epidemiological situation of TB in a particular country.

## Conclusion

This review suggests that there is an unacceptable time delay before the diagnosis of pulmonary tuberculosis is made. There is a need to revise the current case finding strategies under the DOTS strategy adopted world wide. The reported high treatment success rate of directly observed treatment may be supported by measures to shorten the delay in diagnosis. This may result in reduction in transmission and in better tuberculosis control.

## Competing interests

The authors declare that they have no competing interests.

## Authors' contributions

CTS conceived this review, conducted literature search, assessed the studies, carried out the systematic review, and wrote the first draft of the manuscript. KVP assisted in design of review and literature search, assessed the studies, assisted in systematic review and writing initial drafts of the manuscript. JM assisted in data analysis and its interpretation, assisted in writing the methods section and commented on the earlier drafts of the manuscripts. JDVE assisted in the systematic review, manuscript writing, and corrected the initial drafts of the manuscript. All the authors read and approved the final version of the manuscript to be sent for publication.

## Pre-publication history

The pre-publication history for this paper can be accessed here:

http://www.biomedcentral.com/1471-2334/9/91/prepub
